# Accuracy and Test-Retest Reproducibility of Two-Dimensional Knowledge-Based Volumetric Reconstruction of the Right Ventricle in Pulmonary Hypertension

**DOI:** 10.1016/j.echo.2015.02.020

**Published:** 2015-08

**Authors:** Daniel S. Knight, Johannes P. Schwaiger, Sylvia Krupickova, Joseph Davar, Vivek Muthurangu, J. Gerry Coghlan

**Affiliations:** a University College London Medical School, London, United Kingdom; b Department of Cardiology, Royal Free London NHS Foundation Trust, London, United Kingdom; c UCL Centre for Cardiovascular Imaging, University College London, London, United Kingdom

**Keywords:** Transthoracic echocardiography, Right ventricular function, Pulmonary hypertension, Reproducibility of results, Magnetic resonance imaging, CMRI, Cardiac magnetic resonance imaging, FAC, Fractional area change, KBR, Knowledge-based reconstruction, PH, Pulmonary hypertension, RV, Right ventricular, 3DE, Three-dimensional echocardiography, 2D, Two-dimensional, 2DE, Two-dimensional echocardiography

## Abstract

**Background:**

Right heart function is the key determinant of symptoms and prognosis in pulmonary hypertension (PH), but the right ventricle has a complex geometry that is challenging to quantify by two-dimensional (2D) echocardiography. A novel 2D echocardiographic technique for right ventricular (RV) quantitation involves knowledge-based reconstruction (KBR), a hybrid of 2D echocardiography–acquired coordinates localized in three-dimensional space and connected by reference to a disease-specific RV shape library. The aim of this study was to determine the accuracy of 2D KBR against cardiac magnetic resonance imaging in PH and the test-retest reproducibility of both conventional 2D echocardiographic RV fractional area change (FAC) and 2D KBR.

**Methods:**

Twenty-eight patients with PH underwent same-day echocardiography and cardiac magnetic resonance imaging. Two operators performed serial RV FAC and 2D KBR acquisition and postprocessing to assess inter- and intraobserver test-retest reproducibility.

**Results:**

Bland-Altman analysis (mean bias ± 95% limits of agreement) showed good agreement for end-diastolic volume (3.5 ± 25.0 mL), end-systolic volume (0.9 ± 19.9 mL), stroke volume (2.6 ± 23.1 mL), and ejection fraction (0.4 ± 10.2%) measured by 2D KBR and cardiac magnetic resonance imaging. There were no significant interobserver or intraobserver test-retest differences for 2D KBR RV metrics, with acceptable limits of agreement (interobserver end-diastolic volume, −0.9 ± 21.8 mL; end-systolic volume, −1.3 ± 25.8 mL; stroke volume, −0.2 ± 24.2 mL; ejection fraction, 0.7 ± 14.4%). Significant test-retest variability was observed for 2D echocardiographic RV areas and FAC.

**Conclusions:**

Two-dimensional KBR is an accurate, novel technique for RV volumetric quantification in PH, with superior test-retest reproducibility compared with conventional 2D echocardiographic RV FAC.

Right ventricular (RV) function is the key symptomatic and prognostic determinant in pulmonary hypertension (PH).^1^ Cardiac magnetic resonance imaging (CMRI) is the gold standard for volumetric quantification of the right ventricle,^2^ but cardiac ultrasound is a comparatively cheaper and more widely available modality. However, the anatomy and complex geometry of the right ventricle confer significant limitations to two-dimensional (2D) echocardiography (2DE).^3,4^ Fractional area change (FAC), for example, is a simple measure of RV size and function that visualizes only one 2D plane of this complex chamber.^5^ Three-dimensional echocardiography (3DE) has shown promise for RV volumetric analysis in PH^6-8^ but requires operator experience for acquisition and postprocessing beyond that of 2DE, with lower spatial and temporal resolution, typically leading to underestimation of RV volumes.^9^

A novel 2D echocardiographic technique for volumetric RV quantitation involves knowledge-based reconstruction (KBR). This hybrid approach uses the benefits of conventional 2DE in conjunction with a reference library of RV shapes to reconstruct a 3D RV polygon. The feasibility and accuracy of 2D KBR has been demonstrated in a small PH population,^10^ but the ability to accurately identify changes in RV function in response to treatment is also of clinical and prognostic significance.^2,11^ This will depend on the acquisition and postprocessing elements of 2D KBR that both contribute to its variability. We therefore sought to provide further validation data for 2D KBR RV quantification in PH and to investigate the test-retest reproducibility of this novel technique compared with FAC.

## Methods

### Study Population

We performed a prospective cross-sectional study that enrolled 28 patients in sinus rhythm with no contraindications to magnetic resonance imaging who presented for diagnosis and/or follow-up of PH (diagnosed by right heart catheterization as a mean pulmonary artery pressure > 25 mm Hg and a pulmonary capillary wedge pressure < 15 mm Hg^12^). All participants underwent comprehensive 2D transthoracic echocardiography and CMRI on the same day (median scan interval, 116 min; interquartile range, 104–150 min). The etiologies of PH were idiopathic (*n* = 5), connective tissue disease associated (*n* = 14), chronic thromboembolic disease (*n* = 8) and portopulmonary (*n* = 1). Exclusion criteria were arrhythmia and known independent left-sided cardiac disease unrelated to PH.

The study complied with the Declaration of Helsinki. The institutional research ethics committee approved the study, and informed written consent was obtained from all participants.

### Two-Dimensional Echocardiography and KBR

#### Image Acquisition

All patients underwent comprehensive 2D and Doppler transthoracic echocardiography in the left lateral decubitus position using the Philips iE33 echocardiographic system (Philips Medical Systems, Andover, MA) with an S5-1 transducer (frequency bandwidth, 1–5 MHz). A standard clinical protocol for all examinations was followed in conjunction with American Society of Echocardiography guidelines for chamber quantification.^5,13^

**Figure 1:**
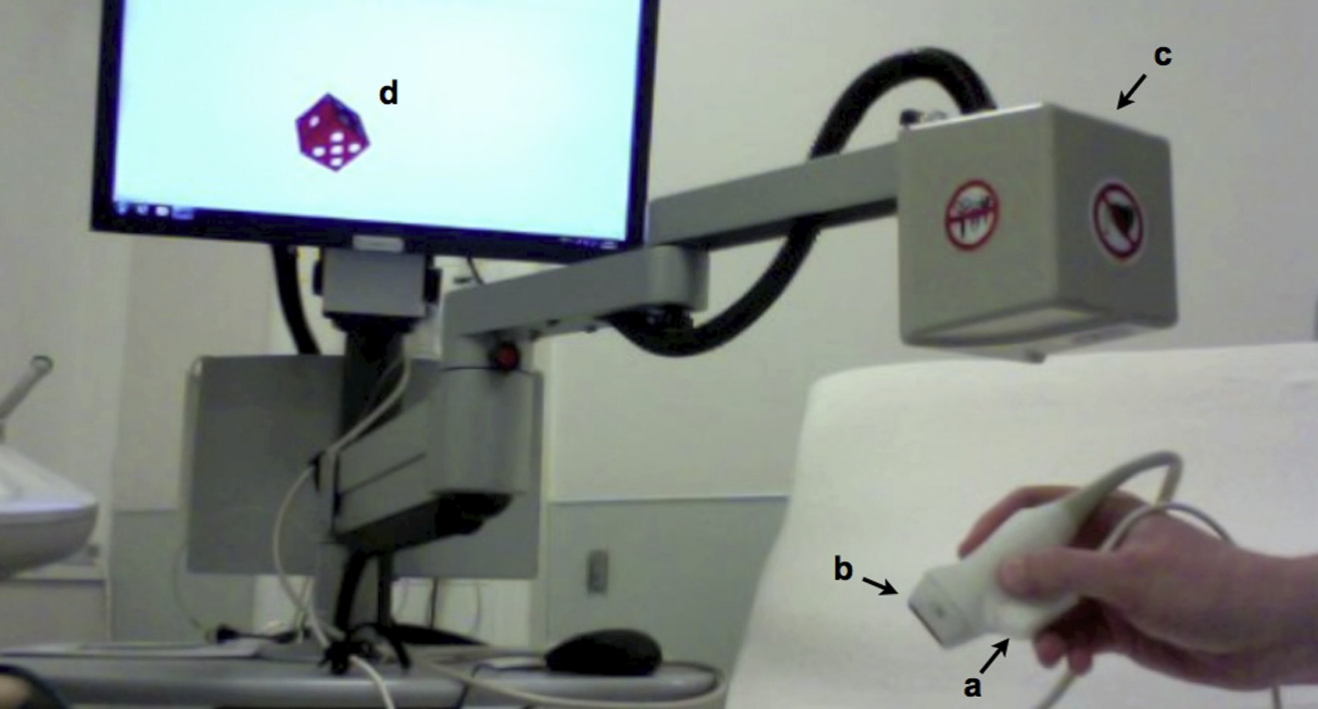
Two-dimensional KBR apparatus: a localizing transducer **(A)** attached by a molded plastic sheath to a conventional 2D echocardiographic probe **(B)** detects orthogonal magnetic fields emitted by the generator **(C)** attached to the mechanical arm that hangs over the patient. Here in the 2D KBR calibration module, the die on the screen **(D)** represents the 2D echocardiographic probe, which moves synchronously with any movement of the 2D echocardiographic probe. Note the cushioned wedge in the background that is placed on the echocardiography couch to ensure that the metallic apparatus underneath couch does not interfere with detection of the magnetic fields.

A magnetic localizer was attached to the S5-1 transducer by a molded plastic sheath. The magnetic localizer was connected to a dedicated console, from which a mechanical arm with an attached magnetic field generator hung over the patient (Figure 1; VentriPoint Diagnostics Ltd, Seattle, WA). The localizer mounted on the ultrasound transducer detects orthogonal magnetic fields from the generator hanging over the patient, and in this manner the ultrasound probe position is localized in 3D space at the point of any 2D acquisition. A cushioned wedge was placed on the echocardiography couch to ensure that the metallic couch apparatus did not interfere with the magnetic field, and patients were instructed to remain entirely stationary in the left lateral decubitus position for the duration of a study acquisition. The ultrasound depth required to visualize all relevant structures was determined before commencing the study and remained fixed throughout.

Seven 2D transthoracic echocardiographic views were obtained in all subjects: parasternal long axis, parasternal short axis at the papillary muscle and apical levels, parasternal RV inflow, parasternal RV outflow including pulmonary valve hinge points and infundibulum, apical four chamber, and an off-axis RV apical view. The 2D KBR acquisition from each view consists of a 2-sec period (usually containing two or three heartbeats) acquired during end-expiratory breath-holds. The electrocardiograph was connected to the echocardiographic system via the dedicated 2D KBR console, and the console images were reproduced from the echocardiographic system’s video output and digitized at 30 frames/sec. Image quality was subjectively graded on a 5-point scale from 0 (very poor) to 4 (perfect).^14^

#### Postprocessing: RV FAC

End-diastolic and end-systolic frames were assigned by visual identification of the largest and smallest RV four-chamber cavity areas, respectively, on the 2D KBR console. These frames were exported to the open-source OsiriX Digital Imaging and Communications in Medicine software for the measurement of RV FAC by tracing the RV endocardium in both frames and using the formula [(end-diastolic area − end-systolic area)/end-diastolic area] × 100.

#### Postprocessing: 2D KBR

**Figure 2:**
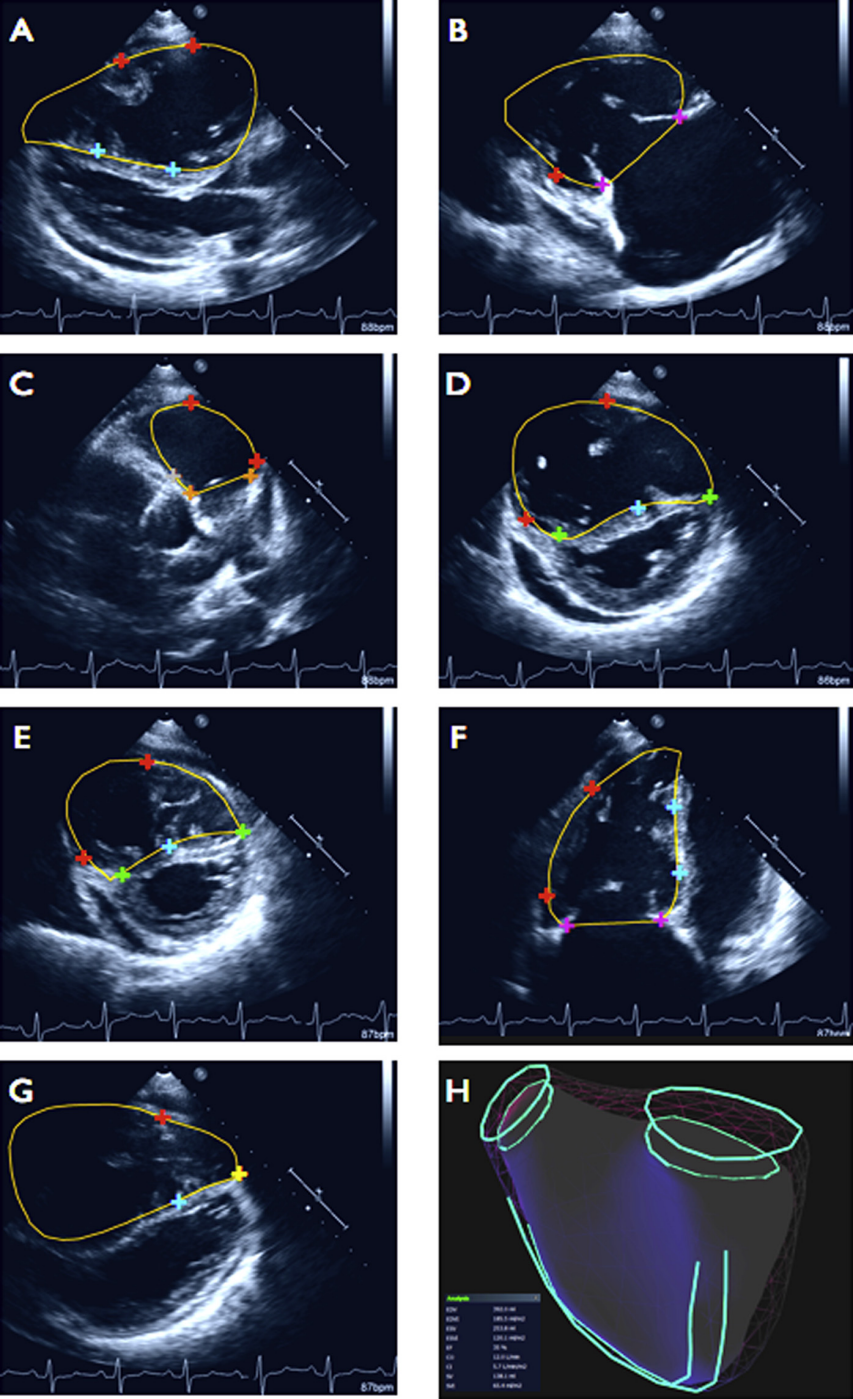
Postprocessed 2D KBR data from a participant with pulmonary hypertension. All of the required 2D echocardiographic scan planes in end-diastole are displayed: **(A)** parasternal long-axis (PLAX), **(B)** PLAX RV inflow, **(C)** PLAX RV outflow including infundibulum and pulmonary valve hinge points, **(D)** parasternal short-axis (PSAX) at midcavity (papillary muscle) level, **(E)** PSAX apical level, **(F)** four-chamber RV, **(G)** off-axis RV apical view (note how the RV apex rides over the left ventricular apex). The *differently colored cross-hairs* represent user-defined plots for different RV structures; for example, *red crosses* are plotted along the RV endocardium, *turquoise crosses* along the RV side of the interventricular septum, a *yellow cross* at the RV apex, *orange crosses* at the pulmonary valve annulus, and *purple crosses* at the tricuspid valve annulus. The *yellow border tracings* are superimposed projections of the 2D KBR RV reconstruction onto the original 2D echocardiographic scan data, also showing how the polygon extends beyond the original 2D echocardiographic image sector. Landmarks can be checked and repositioned by the user if required, and the 2D KBR algorithm subsequently rerun. A final check is the nested view **(H)** of end-diastolic and end-systolic polygons to ensure alignment of the tricuspid and pulmonary valve orifices.

The largest and smallest RV four-chamber cavity areas were visually identified as end-diastole and end-systole, respectively, on the 2D KBR console, with the software subsequently assigning the same time interval between these frames to all other views. On the 2D KBR console, a series of anatomic RV landmarks were identified on the 2D echocardiography images (Figures 2A–2G) in the end-diastolic frames and subsequently in the end-systolic frames. A minimum of 26 points was plotted for each of the end-diastolic and end-systolic data sets. RV endocardial points were placed at the junction between trabeculations and myocardium. The plotted anatomic landmarks with their respective 3D spatial coordinates were then submitted via the Internet to a secure remote server for remote processing by a proprietary 2D KBR algorithm. The algorithm interpolates between the plotted points by referencing against a catalogue of RV shapes generated by CMRI from patients with known diagnoses of PH.

End-diastolic and end-systolic 3D models of the right ventricle were reviewed in a systematic fashion. Intersections between the borders of the 3D model and the original 2D scan plane were inspected to ensure concordance between 2D images and 3D reconstructions (Figures 2A–2G), and marked points were checked for alignment with the surface of the 3D model. Where significant deviations between the reconstructed model and either the plotted points and/or 2D echocardiographic endocardial borders existed, points were replotted and the algorithm was rerun. Where significant border versus 2D image misalignment suggested a shift in patient position or an inadequate breath-hold, all points from that 2D view were removed, and the erroneous 2D echocardiographic view was excluded from the 2D KBR reconstruction. A maximum of one view of the seven required in the data acquisition protocol could be excluded for any given study because of a change in patient position or an inadequate breath-hold. If this problem was encountered in more than one of the seven required views, the entire study was excluded from the final analysis.

The final check entailed inspection of the nested view of end-diastolic and end-systolic models to verify alignment of the tricuspid and pulmonary annular planes (Figure 2H). The final 2D KBR polygon was assessed for precision by subjectively scoring on a 5-point scale depending on the proximity of intersections of the plotted landmarks with the reconstructed polygon: 4 (all points intersect), 3 (three or fewer points significantly deviate from polygon), 2 (five or fewer points significantly deviate from polygon), 1 (seven or fewer points significantly deviate from polygon), and 0 (poor agreement).

#### FAC and 2D KBR Test-Retest Reproducibility

All subjects underwent serial 2D echocardiographic acquisition and postprocessing by two independent sonographers (D.S.K. and J.P.S.), as described previously.^15^ The two sonographers had similar experience in 2D transthoracic echocardiography (>4 years each) and received the same vendor training for the 2D KBR system. Sonographer 1 (D.S.K.) obtained a 2D KBR data set, after which sonographer 2 (J.P.S.) independently obtained a 2D KBR data set. Sonographer 1 then acquired a second 2D KBR data set. The sonographers, who were blinded to each other’s results and the results from CMRI, performed postprocessing of their own data sets for FAC and 2D KBR. Data sets analyzed for intraobserver test-retest reproducibility were postprocessed separately at time intervals of >2 weeks.

### CMRI

#### Image Acquisition

All CMRI images were acquired using a 1.5-T magnetic resonance scanner (Avanto; Siemens Healthcare, Erlangen, Germany) using a 12-element phased-array coil for signal reception and the body coil for signal transmission. A vector electrocardiographic system was used for cardiac gating. In all patients, ventricular volumes and great vessel flow were measured as previously described.^7^ Volumetric RV data were obtained using real-time radial *k*-*t* sensitivity-encoded imaging of contiguous transaxial slices.^16^ Real-time radial *k*-*t* sensitivity-encoded imaging allows the collection of high–spatiotemporal resolution, real-time images during free breathing and is part of the standard clinical CMRI work flow at our institution in the pediatric PH population.^17^

#### Postprocessing

All image postprocessing was performed using “in-house” plugins for the open-source OsiriX Digital Imaging and Communications in Medicine software.^16,18,19^ Endocardial RV borders were traced manually at end-diastole and end-systole, the time points of which were identified by the largest and smallest RV cavity areas, respectively. The inclusion of RV trabeculations was the same as that performed in echocardiographic postprocessing. Ventricular stroke volume was the difference between the end-diastolic volume and end-systolic volume, and ejection fraction was calculated as (stroke volume/end-diastolic volume) × 100.

### Statistical Analysis

Statistical analysis was performed using SPSS version 22.0 (IBM Corporation, Armonk, NY) and Prism version 6.0b for Mac (GraphPad Software, Inc, La Jolla, CA). All continuous data were normally distributed and expressed as mean ± SD. Systematic differences between measurements were evaluated with Student’s paired *t* test (two tailed). *P* values < .05 were considered to indicate statistical significance. Intermodality agreement was studied using the Bland-Altman method, whereby the mean difference was presented as the bias and 95% limits of agreement around the bias expressed as the mean difference ± 1.96 SDs.^20^

Differences between test-retest measurements were analyzed using one-way repeated-measures analysis of variance, with the Bonferroni post hoc test identifying which specific means differed. The Greenhouse-Geisser correction was used if the assumption of sphericity had been violated. Test-retest variability was expressed using intraclass correlation coefficients, relative differences and coefficients of variation. The intraclass correlation coefficient was quantified by the two-way random-effects model with absolute agreement. An intraclass correlation coefficient > 0.85 was considered excellent. Relative differences were calculated by taking the absolute difference between two observations divided by the mean of the repeated observations and expressed as a percentage. Coefficients of variation were calculated as the SD of the difference between two acquisitions divided by their mean value and expressed as a percentage.^21^ A coefficient of variation ≤ 10% was considered excellent.

## Results

### Population Characteristics and 2D KBR Technical Data

The clinical characteristics of the 28 participants are presented in Table 1, all of whom had adequate 2D echocardiographic windows for the specified protocol. Participants’ heart rates recorded on the 2D echocardiographic loop acquired first were similar to those recorded on the 2D echocardiographic loop acquired last (*P* = .90). Image acquisition for one data set took on the order of approximately 5 min per patient, with 2D KBR postprocessing and analysis taking no longer than about 15 min. Good mean subjective scores were observed for 2D echocardiographic image acquisition (2.9 ± 0.9) and 2D KBR reconstruction (3.2 ± 0.7), with moderate correlation between the two scores (*r* = 0.54, *P* = .003).

### RV Quantification by 2D KBR versus CMRI

**Figure 3:**
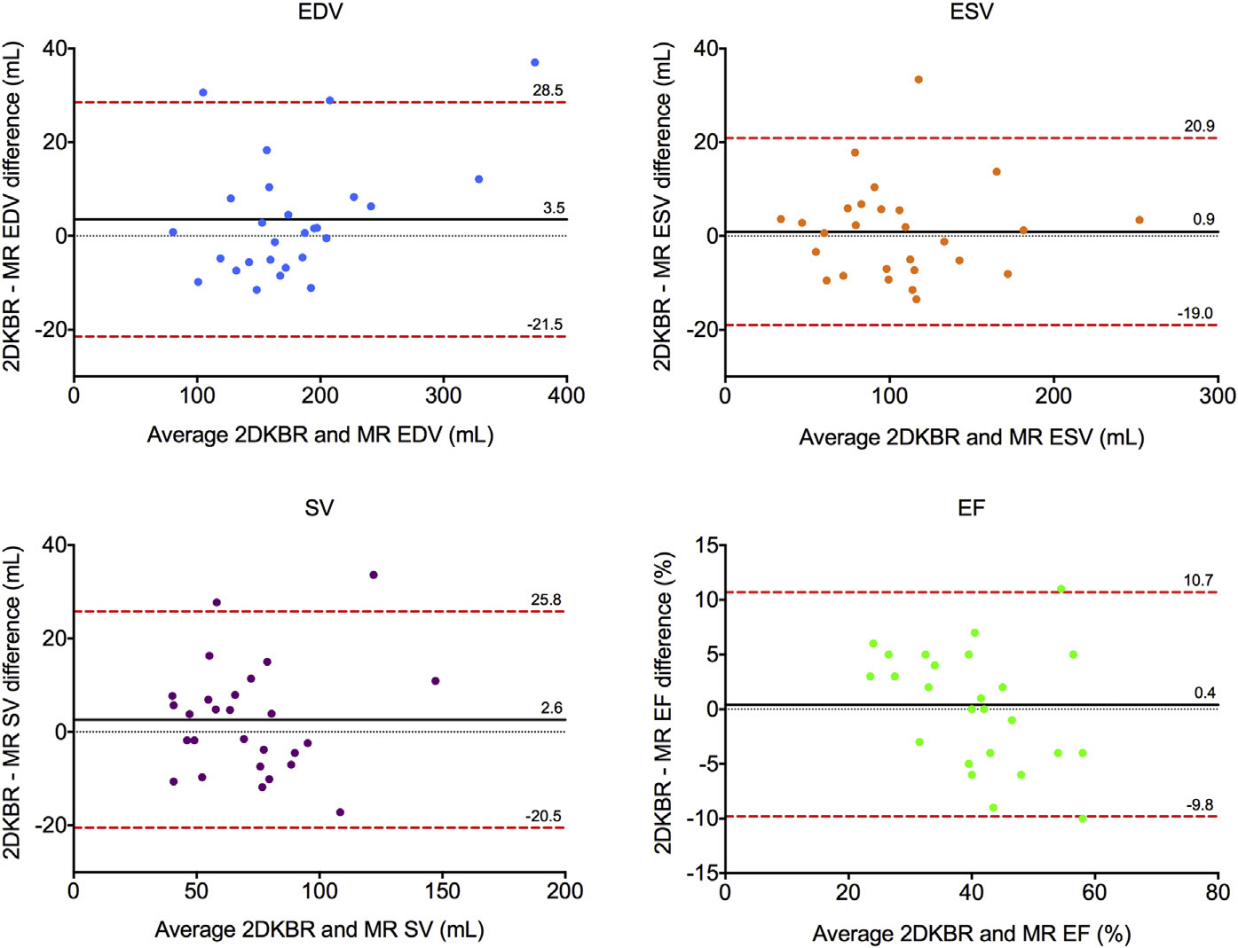
Bland-Altman analysis of bias (*black solid line*) and 95% limits of agreement (*red dashed line*) for 2D KBR versus CMRI quantification of right ventricular end-diastolic volume (EDV), end-systolic volume (ESV), stroke volume (SV), and ejection fraction (EF); *n* = 27 (one patient excluded because of movement artifact during 2D KBR study).

RV volumes and ejection fractions for all participants measured by 2D KBR showed no significant differences with CMRI (Table 2), with no significant bias and clinically acceptable limits of agreement (Figure 3).

### Test-Retest Intraobserver and Interobserver Reproducibility

One patient moved in the first data set acquisition, one patient moved in the third data set acquisition, and two patients moved in both the second and third data set acquisitions. The 2D KBR data sets for these four individuals were therefore excluded from the final test-retest reproducibility analysis because of significant movement artifact.

**Figure 4:**
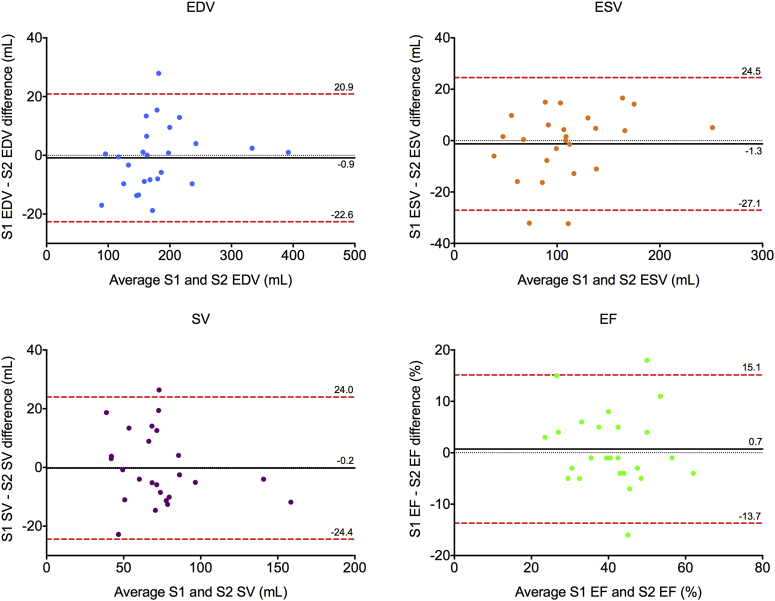
Bland-Altman analysis of bias (*black solid line*) and 95% limits of agreement (*red dashed line*) for interobserver 2D KBR test-retest reproducibility of right ventricular end-diastolic volume (EDV), end-systolic volume (ESV), stroke volume (SV), and ejection fraction (EF); *n* = 25 (three patients excluded because of movement artifact during 2D KBR study). *S1*, Sonographer 1; *S2*, sonographer 2.

Good reproducibility metrics and acceptable limits of agreement were observed for the 24 intra- and interobserver 2D KBR test-retest studies (Table 3, Figure 4). There were no significant differences for RV volumes or ejection fraction between serial 2D KBR studies, but significant intra- and interobserver test-retest variability was demonstrated for serial RV areas and FAC (Table 4).

## Discussion

This study demonstrates the feasibility and accuracy of 2D KBR for RV quantification in PH and provides the first test-retest reproducibility data for this technique. These results suggest a role for 2D KBR in serial follow-up studies of this patient population. The sources of variability at the acquisition and postprocessing stages of 2D KBR have been tested in an approach more akin to clinical practice, with no significant differences demonstrated between serial interobserver and intraobserver test-retest studies. By comparison with conventional RV FAC, 2D KBR has incremental benefit in quantifying RV function through superior test-retest reproducibility.

Two-dimensional KBR is an emerging technique that has been validated in congenital heart disease populations^22-24^ and more recently in a small population of patients with PH.^10^ The utility of applying a hybrid knowledge-based approach to 2DE of the right ventricle is reflected by the known differences in RV shapes that are encountered not only in congenital and acquired disease but also among different subtypes of PH.^25^ Moreover, algorithms for RV reconstruction by conventional 3DE are typically based on generic healthy adult RV shapes rather than taking into account differences in congenital populations or subtle changes in volume- and pressure-overload states.^26^ The reconstruction of a 3D model from 2D landmark coordinates makes the use of the piecewise smooth subdivision surface technique, with gaps between the user-defined points filled by a catalogue registration method that is well validated in vitro.^27,28^ The piecewise smooth subdivision surface technique itself also has greater accuracy over the conventional Beutel method for RV volume reconstruction in vivo by 3DE.^22^

Our limits of agreement are clinically acceptable compared with the gold standard of CMRI, slightly more favorable than those obtained previously in idiopathic PH,^10^ and similar to previous work in children following surgical repair of tetralogy of Fallot.^22^ A potential explanation for these differences might be our quantification of RV volumes by CMRI using a transaxial stack of RV slices rather than the short-axis stack approach. This has the advantage of avoiding partial voluming of the basal RV slices that is of particular relevance in PH because of the relative preservation of longitudinal over radial function.^29^ A transaxial slice orientation facilitates the identification of the inflow and outflow components of the right ventricle and ultimately confers better reproducibility for RV volumetric quantification by CMRI.^30-32^ The 2D KBR hardware used in our study also differs from that in previous studies in terms of the position of the magnetic field generator either above or underneath the patient bed. Our equipment used a magnetic field generator suspended directly above the patient’s chest. However, the magnetic field generator location above or below the echocardiography couch should not theoretically affect the spatial detection of the 2D echocardiographic probe localizer.

To our knowledge, this is the first study designed to assess the test-retest reproducibility of 2D KBR. Importantly, the 2D KBR technique showed no significant differences for interobserver or intraobserver test-retest reproducibility, whereas FAC had significant test-retest variability. The only previous study of test-retest reproducibility of 2D echocardiographic RV area metrics, to our knowledge, had a comparable intraobserver test-retest coefficient of variation for RV FAC of 16.5%.^33^ The reproducibility of FAC postprocessing alone (not including variability in image acquisition) has also been shown to have significant interobserver bias and wide limits of agreement in children after surgical repair of tetralogy of Fallot compared with 2D KBR.^22^ The test-retest reproducibility of 2D KBR RV volumetric quantification is also improved compared with that previously demonstrated by 3DE in either congenital heart disease^15^ or acquired PH.^7^

The superior reproducibility of 2D KBR compared with conventional 2DE and that previously reported for 3DE may be accounted for by several reasons. First, FAC and 3DE require good endocardial delineation to trace the RV border, whereas 2D KBR requires the user to define single points along the endocardium rather than the border in its entirety. Second, in contrast to 2D FAC, the 3D spatial localization of the 2D echocardiographic probe compensates for the acquisition variability in transthoracic windows among operators.^34^ Postprocessing reproducibility is also likely to be enhanced by the KBR process, with our protocol mandating review of the reconstructed models relative to the original 2D echocardiographic pictures. Landmarks are adjusted to ensure acceptable agreement between the raw 2D echocardiographic images and the KBR polygons, thus conferring an element of reproducibility through the KBR algorithm itself. KBR also differs from 3DE by using a shape-specific reconstruction algorithm rather than a generic adult-based algorithm,^26^ hence taking account of the impact of the underlying disease process conferred upon RV morphology.

**Figure 5:**
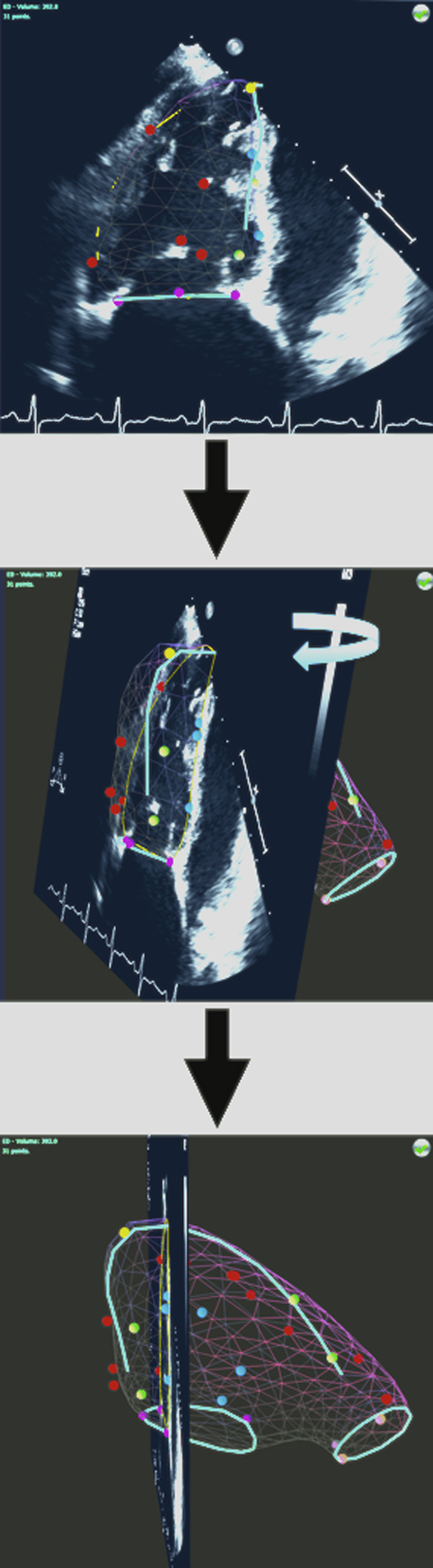
Demonstration of the interaction between the reconstructed 2D knowledge-based reconstruction polygon with a four-chamber view 2D echocardiographic scan plane. The reconstructed polygon can be rotated in any direction (here through 90° from top to bottom, indicated by the curved arrow). Any original 2D echocardiographic acquisition can be displayed (here, the four-chamber view) and viewed in relation to the polygon by clicking on one of the dots. In this way, the reconstructed polygon can be inspected to ensure accurate alignment with the original 2D echocardiographic data. From this view, it is also readily appreciable how much of the right ventricle, predominantly the outflow portion, is neglected in a standard four-chamber view used to derive fractional area change.

Compared with 3DE, the use of 2D echocardiographic technology for data acquisition also has methodologic advantages. Fundamentally, spatial and temporal resolutions of 2DE are higher than those of 3DE. Underestimation of RV volumes is a known limitation of 3DE due to the inferior spatial resolution, conferring blurred endocardial borders and thus a visually smaller RV cavity.^9^ In particular, the contribution from the RV outflow tract is known to be an important determinant of the overall accuracy of RV volumes.^35^ However, accurate visualization of this region can be technically difficult by 3DE.^7,36^ The 2D KBR acquisition protocol includes dedicated imaging of the RV outflow tract by 2DE, affording higher spatial resolution when imaging this region that may contribute to more accurate volumetric quantification (Figure 5). Furthermore, given that echocardiography of the right ventricle has inherent acquisition difficulties due to its anterior position in the chest wall, complex geometry, thin walls, and heavy trabeculations,^3,4^ our subjective image scoring suggests that the requirement for the identification of landmarks rather than the entirety of a cardiac border still permits adequate reconstruction despite cases of poor-quality transthoracic 2D echocardiographic windows.

However, subtle changes in RV function may nevertheless be masked by the margins of error demonstrated in the study. CMRI data demonstrates that a change in RV stroke volume in PH of as little as 10 mL can be regarded as clinically significant,^11^ and therefore 2D KBR may not be able to differentiate minor variations in RV volumes from the variance in reproducibility. CMRI does not have the same acquisition window restrictions and variability inherent to transthoracic echocardiography, with data sets consisting of contiguous fixed-thickness RV slices acquired from the base of the right heart to the main pulmonary artery with the patient in the supine position. A further consideration with respect to the use of 2D KBR in the serial evaluation of patients is that a change in RV volume might confer a change in cavity shape, which could also have implications for the application of the KBR algorithm to follow-up studies.

A disadvantage of 2D KBR is the requirement for several 2D planes to be acquired over separate cardiac cycles. Image acquisition over several cardiac cycles with potential beat-to-beat variability is also a limitation shared by traditional disk summation 3DE and by CMRI, but not with single-beat full-volume 3DE. However, no significant differences were found in heart rates between the start and end of our studies. Acquiring several 2D echocardiographic planes also requires reproducible breath-holding and a stable patient position throughout the study. Repeated breath-holding is also conventionally associated with CMRI, but we used a real-time, high–spatiotemporal resolution sequence as per our institution protocol for PH imaging that allows free breathing and the rapid acquisition of ventricular volumes.^16^ Once image acquisition for a 2D KBR study has commenced, the operator is unable to maneuver the patient to optimize transthoracic echocardiographic windows. Therefore, an optimal patient position for parasternal and apical views must be decided upon before commencing 2D KBR data acquisition. These optimal breath-hold and positional constraints may confer difficulty when applied to acutely unwell individuals, and hence 2D KBR is more likely to be practically applicable in the stable outpatient setting. It should also be remembered that although global volumetric indices of RV function are highly prognostic, they do not account for the heterogeneity in RV regional function in different disease states, as shown by 2D and 3D echocardiographic deformation imaging.^37,38^ Finally, 2D KBR includes the RV trabeculations together with the blood volume, which may in turn affect the accuracy of volumetric indices. However, this is also a limitation shared with 3D echocardiographic techniques and has been shown by CMRI to improve reproducibility metrics compared with excluding trabeculations from the RV cavity volume.^39^

### Limitations

Our study represents a single-center experience with a small participant sample size. However, we have supported the validation data for 2D KBR obtained by previous single-center studies using similar sample sizes,^10,22,23^ and a total of 84 2D echocardiographic studies were performed in our study for test-retest reproducibility purposes. The increase in excluded studies with successive repeated scans was due more to the serial 2D echocardiographic scan acquisition protocol for test-retest reproducibility rather than the 2D KBR technique itself. Only one of 28 patients moved in the first 2D echocardiographic data set image acquisition. Therefore, this limitation is unlikely to be so prevalent for individual clinical scans, and the study analysis times would allow the reacquisition of a second data set within a scheduled clinical echocardiographic examination.

Patients with arrhythmia were specifically excluded from this study, but patients with atrial fibrillation, for example, would require a different approach to 2D KBR post-processing. In atrial fibrillation, the end-diastolic frame for each view would have to be manually selected by visually determining the largest RV cavity size. This could theoretically affect the border alignment of the reconstructed polygon, as different cardiac cycles in separate views will inherently have different end-diastolic volumes because of the variability of irregular R-R intervals. Several reconstructions could be performed on the same data set by selecting different cardiac cycles for each reconstruction, with the resulting 2D KBR RV metrics averaged over the number of cardiac cycles analyzed. However, the accuracy and reproducibility of this approach using 2D KBR in atrial fibrillation requires further investigation.

Finally, CMRI reproducibility data were not acquired in a test-retest format that allowed comparison of the acquisition and postprocessing variability of this technique. However, as detailed above, the acquisition stage of CMRI consists of a set acquisition of cross-sectional, fixed-thickness, contiguous craniocaudal slices that include the entirety of the heart with the patient supine. Therefore CMRI fundamentally has less potential for acquisition variability compared with 2DE, which has imaging windows obtained from different rib spaces acquired in nonuniform patient positions.

## Conclusions

Novel 2DKBR is a feasible and clinically reproducible technique for RV volumetric quantification in PH, with superior test-retest reproducibility compared with 2D echocardiographic FAC for quantifying RV function. It offers the benefits of using operator experience with conventional 2DE for image acquisition and uses algorithmic reconstruction that takes into account the heterogeneity in shape of the RV cavity in different disease states. The applicability of 2D KBR to serial follow-up studies for assessing the response to treatment should be the focus for further work in advancing this novel echocardiography technique.

## Figures and Tables

**Table 1:** Clinical characteristics of study population (*n* = 28)

Variable	Value
Age (y)	54 ± 13
Women	20 (71%)
Height (cm)	165 ± 11
Weight (kg)	71 ± 18
Body surface area (m^2^)	1.8 ± 0.3
Heart rate (beats/min)	79 ± 13
Mean PASP on RHC (mm Hg)	47 ± 12
Pulmonary vasodilators	
Endothelin antagonists	11 (39%)
PDE_5_ antagonists	18 (64%)
Oral prostanoid	1 (4%)
Inhaled prostanoid	1 (4%)
RV EDV (mL/m^2^)	98 ± 26
RV ESV (mL/m^2^)	59 ± 23
RV EF (%)	41 ± 11

*EDV*, End-diastolic volume; *EF*, ejection fraction; *ESV*, end-systolic volume; *PASP*, pulmonary artery systolic pressure; *PDE*_*5*_, phosphodiesterase 5; *RHC*, right heart catheterization. Data are expressed as mean ± SD or as number (percentage). RV volumes are derived from cardiac magnetic resonance imaging.

**Table 2:** RV volumes and EF by 2D KBR versus CMRI

Measurement	2D KBR	CMRI	*P*∗
RV EDV (mL)	179 ± 66	176 ± 61	.16
RV ESV (mL)	107 ± 47	106 ± 47	.63
RV SV (mL)	73 ± 27	70 ± 26	.26
RV EF (%)	42 ± 10	41 ± 11	.66

*EDV*, End-diastolic volume; *EF*, ejection fraction; *ESV*, end-systolic volume; *SV*, stroke volume. Data are expressed as mean ± SD; *n* = 27 (one patient excluded because of movement artifact during 2D KBR study).

∗ Paired Student’s *t* test.

**Table 3:** Test-retest reproducibility results for 2D KBR and 2D echocardiographic RV metrics

Variable	Intraobserver			Interobserver		
	ICC	COV (%)	RD (%)	ICC	COV (%)	RD (%)
2D KBR						
RV EDV	0.985	3.0	4.2	0.986	3.9	5.5
RV ESV	0.987	4.3	6.1	0.960	7.7	10.9
RV SV	0.953	8.3	11.7	0.856	11.7	16.5
RV EF	0.919	6.4	9.0	0.758	10.5	14.8
2DE						
RV EDA	0.885	9.0	12.7	0.394	25.0	35.4
RV ESA	0.931	9.3	13.2	0.440	31.0	43.9
RV FAC	0.784	18.1	25.6	0.619	20.8	29.4

*COV*, Coefficient of variation; *EDA*, end-diastolic area; *EDV*, end-diastolic volume; *EF*, ejection fraction; *ESA*, end-systolic area; *ESV*, end-systolic volume; *ICC*, intraclass correlation coefficient; *RD*, relative difference; *SV*, stroke volume.

**Table 4:** Interobserver and intraobserver test-retest reproducibility of RV volumes and EF by 2D KBR and RV areas and FAC by 2DE

Variable	Sonographer 1.1	Sonographer 2	Sonographer 1.2	*P*∗
2D KBR				
RV EDV (mL)	184 ± 68	185 ± 65	180 ± 66	.17
RV ESV (mL)	110 ± 49	111 ± 45	111 ± 48	.80
RV SV (mL)	74 ± 27	74 ± 30	69 ± 27	.15
RV EF (%)	41 ± 10	41 ± 11	40 ± 10	.39
2DE				
RV EDA (cm^2^)	23 ± 6	32 ± 7	23 ± 7	<.001
RV ESA (cm^2^)	15 ± 6	22 ± 6	16 ± 6	<.001
RV FAC (%)	36 ± 15	31 ± 10	34 ± 14	.05

*EDA*, end-diastolic area; *EDV*, end-diastolic volume; *EF*, ejection fraction; *ESA*, end-systolic area; *ESV*, end-systolic volume. Data are expressed as mean ± SD; *n* = 24 for 2D KBR (four patients excluded because of movement artifact), *n* = 27 for FAC (one patient had an unanalyzable four-chamber image that precluded FAC but not 2D KBR).

∗ One-way repeated measures analysis of variance.
